# A Stackelberg Security Game for Adversarial Outbreak Detection in the Internet of Things

**DOI:** 10.3390/s20030804

**Published:** 2020-02-01

**Authors:** Lili Chen, Zhen Wang, Fenghua Li, Yunchuan Guo, Kui Geng

**Affiliations:** 1 State Key Laboratory of Integrated Services Networks, Xidian University, Xi’an 710071, China; chenlili_iie@163.com (L.C.); lifenghua@iie.ac.cn (F.L.); 2 Institute of Information Engineering Chinese Academy of Sciences, Beijing 100093, China; wangzhen@hdu.edu.cn (Z.W.); guoyunchuan@iie.ac.cn (Y.G.); 3 School of Cyberspace, Hangzhou Dianzi University, Hangzhou 310018, China; 4 School of Cybersecurity, University of Chinese Academy of Sciences, Beijing 100049, China

**Keywords:** outbreak detection, Internet of Things, Stackelberg game, dynamic scheduling strategy

## Abstract

With limited computing resources and a lack of physical lines of defense, the Internet of Things (IoT) has become a focus of cyberattacks. In recent years, outbreak propagation attacks against the IoT have occurred frequently, and these attacks are often strategical. In order to detect the outbreak propagation as soon as possible, t embedded Intrusion Detection Systems (IDSs) are widely deployed in the IoT. This paper tackles the problem of outbreak detection in adversarial environment in the IoT. A dynamic scheduling strategy based on specific IDSs monitoring of IoT devices is proposed to avoid strategic attacks. Firstly, we formulate the interaction between the defender and attacker as a Stackelberg game in which the defender first chooses a set of device nodes to activate, and then the attacker selects one seed (one device node) to spread the worms. This yields an extremely complex bilevel optimization problem. Our approach is to build a modified Column Generation framework for computing the optimal strategy effectively. The optimal response of the defender’s problem is expressed as mixed-integer linear programming (MILPs). It is proved that the solution of the defender’s optimal response is a NP-hard problem. Moreover, the optimal response of defenders is improved by an approximate algorithm--a greedy algorithm. Finally, the proposed scheme is tested on some randomly generated instances. The experimental results show that the scheme is effective for monitoring optimal scheduling.

## 1. Introduction

### 1.1. Background

With the rapid development of communications technology and perceptual recognition technology, the Internet of Things [1] has been widely applied in intelligent medical treatment, intelligent transportation and other areas, which has brought tremendous convenience to peoples’ lives [2], but lots of security challenges at the same time. In recent years, the IoT has been subjected to a large number of attacks with typical outbreak propagation features [3]. Through outbreak propagation, a worm can infect a wide range of devices (e.g., intelligent devices, smart webcams) in a short period [4], resulting in severe economic losses, and even large-scale network paralysis. For example, in October 2016, the Mirai worm [5] infected some 500,000 IoT devices on the east coast of the United States within 6 hours, causing DYN to lose $110 million; in May 2017, the WannaCry worm attacked more than 300,000 users in 150 countries in 24 hours, causing a loss of 8 billion US dollars [6], especially for users with IoT devices, such as the medical systems [7]. To detect the worm outbreak propagation as soon as possible, we can collect potential threat data by deploying specific embedded Intrusion Detection Systems (IDSs) in the IoT [8,9]. Therefore, the rapid detection of the worm outbreak propagation in the IoT with limited resources by IDS has become an urgent problem to be solved. Herein, such IoT devices with embedded IDS are collectively referred to as “sensors”. The "scheduling strategy" mentioned in the following chapters of this paper is mainly aimed at methods of “sensor” combination opening.

### 1.2. Motivation

Generally, the initiator of outbreak propagation is a malicious party, and our goal is to detect the outbreak propagation as soon as possible. For example, in a water network, the problem is where should we place a limited number of sensors to quickly detect contaminants [10,11,12,13,14], while in smart power grids, what is concerned about is finding the failure node in a short time [15]. It is natural in these scenarios to consider the problem of adversarial outbreak detection (AOD), where the defender can first select a set of nodes to deploy sensors, and subsequently the attacker can choose the source of infection to spread malicious information. Although there have been many studies on the AOD problem [11,15], the scenario setting of these works (e.g., a water network setting) is different from our scenario and assumptions (e.g., cybersecurity settings and parameters). A common assumption in the existing AOD problem is that the attacker first chooses an initially infected node according to a probability distribution [10,12]. However, in our scenario, the attacker strategically initiates an outbreak propagation by considering the monitoring strategy in the IoT. Therefore, the following problems need to be considered for our scenario: 

#### 1.2.1. The Uncertainty of Propagation Trajectory

**Figure 1:**
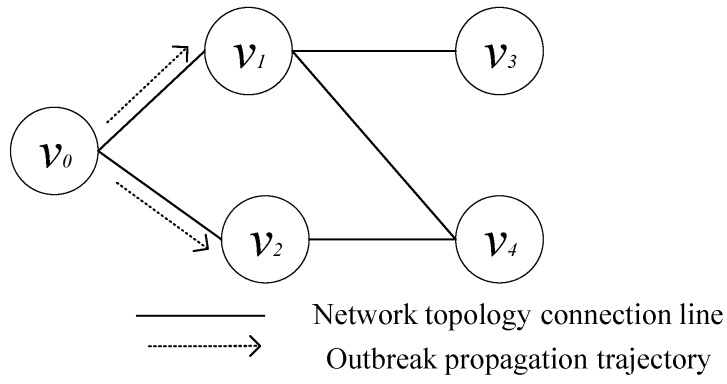
Propagation Trajectory.

The propagation trajectory is uncertain. For instance, the attacker exploits a vulnerability to infect node $v_{0}$, which has two adjacent nodes $v_{1}$ and $v_{2}$. There are some vulnerabilities on nodes $v_{1}$ and $v_{2}$ that can be exploitable by attackers, respectively. It is uncertain which vulnerability on nodes $v_{1}$ and $v_{2}$ will be exploited by the attacker to spread malicious code, as shown in Figure 1. The uncertainty of the outbreak propagation trajectory leads to two problems: (1) the outbreak propagation process is dynamic; (2) the outbreak detection time is uncertain. It should be considered how to simulate the dynamic of the outbreak propagation process, and how to determine the time when outbreak propagation is detected. Moreover, the determination of detection time will be complicated by the propagation dynamics and uncertainty.

#### 1.2.2. The Limit of Computing Resource 

Most of the devices in the IoT are micro-embedded, with limited hardware and software resources as well as minor computing tasks, and more resources need to be saved for normal services. For example, in smart grid defense, it may not be realistic to apply the latest patch to all systems since it may potentially require system downtime affecting customer service [15]. Continuous running of the IDS program can cause the excessive power consumption which will lead to the crash of the IoT network. Thus, the balance between limited resources and scheduling monitoring needs to be considered.

#### 1.2.3. The Dynamics of Scheduling Strategy

The IoT system is composed of a mass of devices that are frequently exposed to public situations, lacking effective protection, and are more vulnerable to attack [16]. For example, while the administrator adopts a regular scheduling strategy to monitor network security status, the attacker can obtain the relevant information (e.g., position) of the sensors through some attack analysis tools (such as eavesdropping [17], extended sleep [18], polymorphic worm [19], probe-response attack [20]). The attacker strategically selects a network device node as a source of infection outbreak propagation through acquired sensors information to avoid being monitored [20]. There have been some existing works taking into account the adversarial environment [10,11,15], but the impact of the attacker on monitoring scheduling strategy has been neglected. Due to the certain regularity of the existing schemes and it cannot deal with the above attacker’s strategical attack.

In view of the above three aspects, there are many studies. The principles of epidemic modelling were applied to IoT networks composed of wireless sensor nodes to describe the uncertain propagation trajectory, where the most commonly are model SI, SIR and SIS [21,22,23]. The game theory was introduced into IoT network to obtain allocated resources for the defenders and the attackers [11,16,24,25,26]. The adversary was set as the attacker chooses to pollute when knowing the position of the sensor, and some deployment schemes of sensors in the adversarial environment was proposed [11,27,28]. While, under such circumstance, it is considered that the robustness of collecting threat data and detecting outbreak propagation can be affected by the strategic attack. Thus, existing approaches cannot be directly applied in current situation. It is necessary to design an effective scheduling strategy to carry out irregular dynamic monitoring of adversarial outbreak propagation in IoT, making the results of detection more robust.

### 1.3. Our Contribution

To address the above challenges, we used the Monte-Carlo [29] method to simulate the outbreak propagation process of trajectory uncertainty. We built the AOD problem as a Stackelberg model, in which the controller of the IDSs acts as the defender, first selects the scheduling strategy (i.e., the initial strategy), the initiator of the outbreak propagation as the attacker, and then the source node of the outbreak propagation based on the selected open sensors selected by the observed defender. It is more realistic considering the impact of attackers on monitoring scheduling strategy, aiming at the attack and defense process between administrators and attackers. The leader (defender) will select several nodes of the network for monitoring, whereas the follower (attacker) will select one node in the network as the source of infection. The contribution of this paper is as follows:

- Firstly, we generated a “Propagation Time Initialization Table” to obtain the determined detection time.
- Secondly, we built a Stackelberg model which is proposed to formulate the Adversarial Outbreak Detection problem in limited resource for IoT.
- For the Stackelberg model, we adopt a CG-AOD algorithm. As the response of defender is an NP-hard problem, we formulated mixed-integer linear programming (MILPs) and exploited an approximate algorithm--greedy algorithm to speed up the solution. Theoretically, we proved that an approximate ratio of constant factors can be obtained through the algorithm.
- Finally, we design a numerical experiment and a series of experiments in terms of solution time and quality by constructing a specific network case model and expansion model. The results show that the CG-AOD and CG-AOD/Appro algorithm is robust and scalable.

The rest of this paper is organized as follows: In Section 2, we discuss related work. In Section 3, we introduce our theoretical model. Section 4 formulates CG-AOD algorithm as two parts and analyzes the corresponding factors. Experiments and the analysis are given in Section 5. A conclusion is drawn in Section 6.

## 2. Related Work

We divide the related work into four parts: (i) IDS for IoT (ii) outbreak detection, (iii) the use of game theory in computer security, and (iv) Monte Carlo method in IoT.

### 2.1. IDSs for IoT 

In an IoT network, due to the complex environment, the different standards and communication stacks involved, the limited resources, as well as lack of a physical line of defense (i.e., there are no gateways or switches to monitor the information flow), traditional IDS techniques are difficult to apply. Current IDSs for IoT technologies is focused on the architecture type, and from this point of view, the future development direction of IoT IDS is proposed and evaluated [30]. Zarpelao [8] classified the IDSs into four attributes which include detection method, IDS placement strategy, security threat and validation strategy, and introduced the work of making specific IDS schemes for IoT or developing an attack detection strategy that may be embedded in IDSs. In addition, to promote multi resource sharing and heterogeneous resource demand allocation, Lin [16] recommended an IDS architecture and a resource allocation scheme with edge computing. Sforzin [31] proposed architecture based on limited resource containing devices which can be effectively serve as IDS in IoT. These works have indeed taken into account of the deployment of IDSs, but no dynamic scheduling strategy based on IoT device specific IDSs are involved.

### 2.2. Outbreak Detection 

The second part of work is outbreak detection, which involves deploying a limited number of sensors to detect the spread of an outbreak propagation as early as possible. In previous schemes, the outbreak detection improves the efficiency and accuracy of the algorithm from the perspective of the sensor by exploiting sub-modularity to obtain an approximation value (1-1/e), but not take into account the strategic behavior of the attacker and ignores the setting of the adversarial environment. Obviously, the IoT device without the physical line of defense becomes a target of interest for the attacker. The related works of AOD problem are as follows: Leskovec [10] firstly studied outbreak detection problem and gave an efficient algorithm—Cost-Effective Lazy Forward (CELF) selection—by exploiting sub-modularity which is 700 times faster than a simple greedy algorithm. Many works are proposed to improve the CELF algorithm [22,32,33,34]. Krause [35] first considered the setting of an adversarial environment and proposed a SATURATE algorithm to ensure that the solution is superior to the current best solution, but required a higher cost than CELF. However, this work is focused on a rather static scenario, where the adversary first chooses an initially infected node according to a probability distribution, which is fundamentally different from our case. 

### 2.3. Security Game 

The third part of work is a security game that has been used to calculate the optimal allocation of security resources in IoT under an adversarial environment. In view of the traditional game model, there are many deficiencies, such as analysis of complexity, slow convergence speed and the high cost of exchange of information/problems. Semasinghe [36] discussed the multiple uncommon game theory model (e.g., evolutionary game, the average field game, and so on), so as to adapt to the inherent characteristics of IoT network in the future. For the resource allocation problem in IoT, Rullo [37] proposed a linear programming based formulation for solving the problem of optimally placing a set of security resources in static IoT networks, considering a fixed defender budget, the strategic behavior of the attacker, and modeling the interaction between attacker and defender as a Stackelberg game. Altman [38] proposed a game theory-based approach to study jamming attacks in wireless networks. Nmavar [39] distributed the power of the subcarriers to decrease the aggregate bit error rate (BER) for thwarting the jamming attack. Zhu [40] investigated sensor networks by a multi-player game which is formalized to model the non-cooperative strategic behavior between the attackers and the network. Cheng [41] proposed the approach based clustering algorithm and used Pareto to analysis the optimal resource management from the perspective of game theory. In addition, there are some models very similar to the adversarial environment setting in this work, and two categories based on the difference in the player’s strategy space: double oracle [42,43,44] and column generation [45,46,47]. The work provided some methods to optimize the deployment of the sensor for outbreak detection problem, but the scenario setting of these methods are different from ours.

### 2.4. Monte Carlo Method in IoT 

In the field of IoT security, Monte Carlo method is applied to several scenarios. In the mobile Internet of Things scenario, Rullo [48] addressed the problem of security resource allocation by using the Monte Carlo method to generate different device configurations in a geographic area in order to evaluate the proposed method with several network topologies. Xu [17] focused on the transmission design for secure relay communications in IoT networks, where the communication is exposed to eavesdroppers with unknown number and locations, and exploited Monte Carlo model simulations to validate the theoretical result.

The aforementioned work provided some methods to optimize the deployment of sensor for the outbreak detection in IoT and many security games methods based on resource-limited for deploying sensors. However, compare with our work, the scenario setting of these methods, including threat propagation process, utility calculation methods, and so on, are totally different.

## 3. Adversarial Outbreak Detection Game Model

In this paper, we introduce the attacker factor into the AOD problem and formulate the problem as a Stackelberg game for IoT. In our Stackelberg game, the network manager is the leader who moves first to perform the dispatch of the sensor, and the attacker is the follower who observes the strategy of the leader and responds to it.

**Figure 2:**
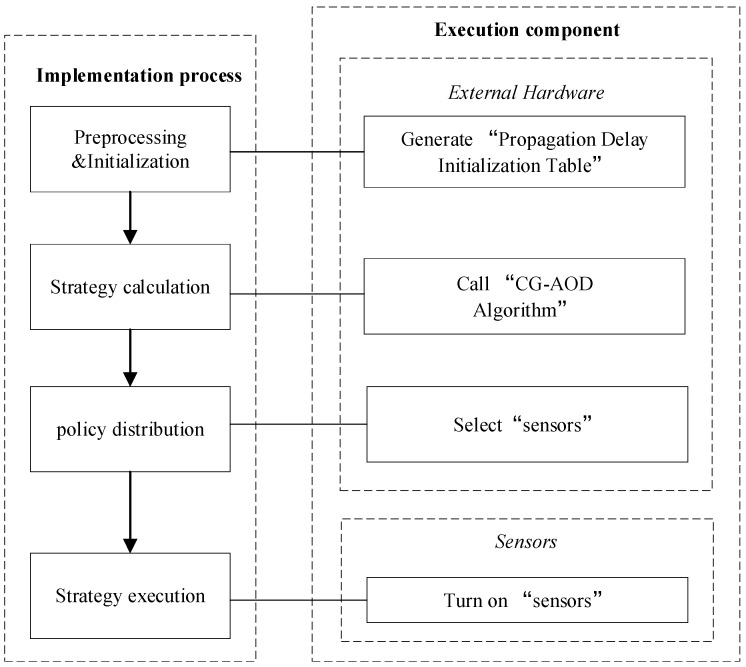
Implementation Process Framework.

In order to clearly describe the whole process of project implementation, the Implementation Process Framework is given in Figure 2, which consists of four stages: preprocessing and initialization, strategy calculation, policy distribution, and strategy execution. In external hardware, the “Processing Time Initialization Table” is generated, the “CG-AOD Algorithm” is called, the signal of schedule is sent. In sensors, the sensor is activated.

### 3.1. Network

We formulate the communication network deployed with sensors as an undirected graph G(V,E) (called a target network), where v∈V is the node and e∈E represents the connection between two nodes. In this graph, each node v∈V can deploy with a sensor, and it can also be the candidate for the attacker. Par(v)) is the set of the parent node of v. Furthermore, each edge e∈E has a label denoted by p:E→(0,1] to describe the probability of infection propagation through it. For each edge e=(u,v)∈E, p(u,v) represents the probability that v is infected by u through the edge (u,v), while if (u,v)∉E, p(u,v):=0. Although the structure of the target network varies over time, the critical devices in it, which are often the main targets of attackers, do not change their position frequently after the collector deployed. In other words, the network structure composed of nodes of the deployed collector is stable. Hence, we assume that the network structure is static.

### 3.2. Virus Propagation

Considering that our work is only to collect IoT device data and detect IoT device state, we exploit the discrete-time infection SI model [22], which is consist of the Susceptible(S) and Infected(I). The SI model used to simulate the outbreak propagation in IoT can be described as follows: Let $I_{t}\subseteq V$ be the set of nodes that get infected at the step t≥0 with $I_{0}=u$. We define $J_{t}:=\cup_{0\leq i\leq t}I_{i}$ as the cumulative set of nodes that get infected before step t≥0. Then, at step t+1, each infected node $u\in J_{t}$ may infect its out-neighbors $v\in V\backslash J_{t}$ with an independent probability of p(u,v). Thus, a node $v\in V\backslash J_{t}$ is infected at step t + 1 with the probability:

(1) $$1-\prod_{u\in J_{t}\cap Par\left(v\right)}\left(1-p\left(u,v\right)\right)$$

If node v is infected successfully, it will be added into the set $I_{t+1}$. Then $J_{t+1}$ will be updated by $J_{t+1}\leftarrow J_{t}\cap I_{t+1}$. Note that each infected node has more than one chance to activate its susceptible out-neighbors until they get infected, and each node keeps its infected state once it is infected by others. The cumulative infected process ${\left(J_{t}\right)}_{t\geq 0}$ is Markovian.

### 3.3. Strategies 

A pure defender strategy $D=\langle D_{v}\rangle$ is to select k sensors to monitor, i.e., $\sum_{v\in V}D_{v}=k,$ where $D_{v}\in \left\{0,1\right\},\;D_{v}$ = 1 indicates that the sensors v are opened to monitoring; otherwise, $D_{v}$ = 0 indicates that the sensors v is not opened to monitoring. In other words, the vector D consists of |V| elements, only k of which are 1, and the others are all 0. The defender’s pure strategy space is denoted by D, in which there are (|V|, *k*) pure strategies. $V_{D}$ represents a collection of all sensors in strategy D. A mixed defender strategy is a probability distribution over pure strategies, i.e., $x=\langle x_{D}\rangle$ with $x_{D}$ representing the probability that D is played, where, $\sum_{D\in D}x_{D}$ = 1, the sum of all $x_{D}$ is equal to 1. In other words, each pure strategy D corresponds to a probability $x_{D}$, and the number of $x_{D}$ equals to the number of the pure strategies, that is, (|V|, *k*). Note that the number of $x_{D}$ that are not 0 cannot be limited, but usually less than (|V|, *k*).

The attacker choose only one node from all nodes in IoT as the source of infection, which is a pure strategy for the attacker. We denote an attacker’s pure strategy as a vector $A=\langle A_{v}\rangle$, where $A_{v}$ = 1 if v∈V, and the node is selected as the source of infection by the attacker; otherwise, $A_{v}$ = 0. In other words, vector A consists of |V| elements, only one of which is 1, and the others are all 0. The attacker’s pure strategy space is denoted by A, in which there are |V| pure strategies. $V_{A}$ represents a collection of all nodes in strategy A. The attacker’s mixed strategy is denoted by $y=\langle y_{A}\rangle$ with $y_{A}$ representing the probability that A is played.

### 3.4. Utility

As most related works do, we formulate the issue of activating *k* optimal sensors as a zero-sum game. The defender wants to detect the attacker’s infected node as early as possible, while the attacker tries to keep the period of infection propagation as long as possible. It should be noted that when an infected node is detected, the process of spreading all malicious information in the target network is terminated immediately. 

Given defender’s strategy set D and attacker’s strategy set A, if D∩A=∅, defender’s strategy and the attacker’s strategy select sets of nodes with no intersection, which means that the defender fails in detecting the infection event, the attacker succeeds, he will gain a payoff P(A)=−P(D). Otherwise, if D∩A=0, which means that the defender catch the attacker, the defender’s payoff is P(D) = −P(A). Due to the probability of infection, it is uncertain whether node v∈D can be successfully infected by the parent node Par(v). Therefore, the Monte Carlo method is adopted to simulate the infection process for MC times and record every result of each time. We store these records in the “Propagation Time Initialization Table”, as shown in Table 1. The “ Propagation Time Initialization Table” records the time when each node $u_{A}\in V$ in the network successfully infects other nodes as the source node, and the record of the simulation once. In addition, the “Propagation Time Initialization Table” is an offline table, which is generated in the preprocessing phase in Figure 2. After the preprocessing phase is generated, the “Propagation Time Initialization Table” is stored in external hardware in the form of files, which can be directly read for obtaining in subsequent use. 

The time spent in the process of propagation from the infected node $u_{i}\in V$ to all the nodes $v_{j}\in V$ in the network can be defined as follows:

(2) $$\alpha \left(u_{i},v_{j}\right)=\alpha \left(i,j\right)=\{\begin{array}{cc} 0, & i=j\\ N, & i\neq j \end{array}$$

where N is a positive integer.

According to the ” Propagation Time Initialization Table”, the time of an attacker’s pure strategy A being detected by the defender’s pure strategy D in the Monte Carlo process is defined as:

(3) $$t\left(A,D\right):=\min\left\{\min\left\{\alpha \left(u_{i},v_{j}\right)|I_{t}\cap V_{D}\neq \emptyset \;with\;I_{0}=V_{A}\right\},\;T_{max}\right\}$$

where $T_{max}$ is the time interval that we observe.

Since the malicious information successfully infects the neighboring node with a certain probability, using the Monte Carlo method, the detection time τ(A,D) indicates the time that a pure strategy *A* of the attacker performs the malicious information propagation is detected by one of the sensors in the defender’s pure strategy *D*:

(4) $$\tau \left(A,D\right):=\frac{1}{MC}\overset{MC}{\underset{m=1}{\sum }}t_{m}\left(A,D\right)$$

The defender’s payoff function is as follows:

(5) P(D)=τ(A,D)

Attacker’s payoff function is P(A)=−P(D).

The mixed strategy function of the attacker is as follows: 

Given a defender’s mixed strategy x and an attacker’s pure strategy *A*, the expected attacker utility is:

(6) $$U_{a}\left(x,A\right)=P\left(A\right)\underset{D\in D}{\sum }\left(1-z_{D,A}\right)x_{D}$$

where $z_{D,A}$ means that defender’s strategy x overlaps attacker’s strategy A. It should be noted that z is the flag bit here, which is used to facilitate the reader to understand the meaning of the formula, that is, z = 1 if D∩A≠∅; z=0 if D∩A=∅. In the following chapters, the meaning of this flag bit has been included in the objective function and constraints, so it does not appear clearly. 

Similarly, the attacker’s expected utility $U_{a}\left(D,y\right)$ of playing mixed strategy y against *D* is:

(7) $$U_{a}\left(D,y\right)=\underset{A\in A}{\sum }\left(1-z_{D,A}\right)y_{A}\;P\left(A\right)$$

When both players use mixed strategies, the expected utility of the attacker is:

(8) $$U_{a}\left(x,y\right)=\underset{D\in D}{\sum }x_{D}U_{a}\left(D,y\right)=\underset{A\in A}{\sum }y_{A}U_{a}\left(x,A\right)$$

The mixed strategy function of the defender is as follows: 

Given a defender’s mixed strategy x and an attacker’s pure strategy A, the expected attacker utility is:

(9) $$U_{d}\left(x,A\right)=\underset{D\in D}{\sum }\left(1-z_{D,A}\right)x_{D}\;P\left(D\right)$$

where, the parameter z in defender’s function is the same as the parameter z in attacker’s function, which will no more details here.

The defender’s expected utility $U_{d}\left(D,y\right)$ of playing mixed strategy y against D is:

(10) $$U_{d}\left(D,y\right)=P\left(D\right)\underset{A\in A}{\sum }\left(1-z_{D,A}\right)y_{A}$$

When both players use mixed strategies, the expected utility of the attacker is:

(11) $$U_{d}\left(x,y\right)=\underset{D\in D}{\sum }x_{D}U_{d}\left(D,y\right)=\underset{A\in A}{\sum }y_{A}U_{d}\left(x,\;A\right)$$

### 3.5. Equilibrium

The Stackelberg equilibrium (SSE) is equal to the Nash equilibrium under the given zero-sum assumption (the defender maximizes the minimum utility, or equivalently, minimizes the maximum attacker utility). Thus, the optimal mixed strategy x of both players can be computed by solving the following linear programming (LP) problem:

(12) max          U

(13) $$s.t.\;\;U\leq U_{d}\left(x,A\right)\;\forall A\in A$$

(14) $$\sum_{v\in V}D_{v}=k$$

(15) $$\sum_{D\in D}x_{D}=1$$

(16) $$x_{D}\geq 0\;\forall D\in D$$

Equation (12) is the optimization goal. Equation (13) enforces that U is the minimum utility. Equation (14) enforces that the defender covers a maximum of *k* nodes. Equation (15) ensures that the summation of all mixed strategies equal to 1. Equation (16) guarantees that probability of the mixed strategy being chosen is not 0.

When strategy spaces of both players are small, the optimal solution can be obtained by solving the LP. However, with the growing scale of the network |V|, the attacker’s strategy space increases, and the space of the defender’s strategy grows exponentially with the budget *k*, thus the LP cannot find the optimal solution in a short time. A new algorithm is required to solve the optimal strategy of both sides.

## 4. Approach

In order to solve the problem in the previous section, the idea of column generation algorithm is introduced [15]. The main idea of the column generation algorithm is to divide the problem into the *Optimal Response* and the *Approximation Response*, and the new variable added into the *Approximation Response* will maximize the value of *Optimal Response.* The algorithm terminates when the *Approximation Response* cannot identify any new variables that can improve the value of *Optimal Response*. The column generation algorithm can effectively reduce the time to obtain the optimal solution when the strategy space of one of the two sides in the game is large. The overall framework of the algorithm is presented in the following section.

### 4.1. Overview of CG-AOD

**Figure 3:**
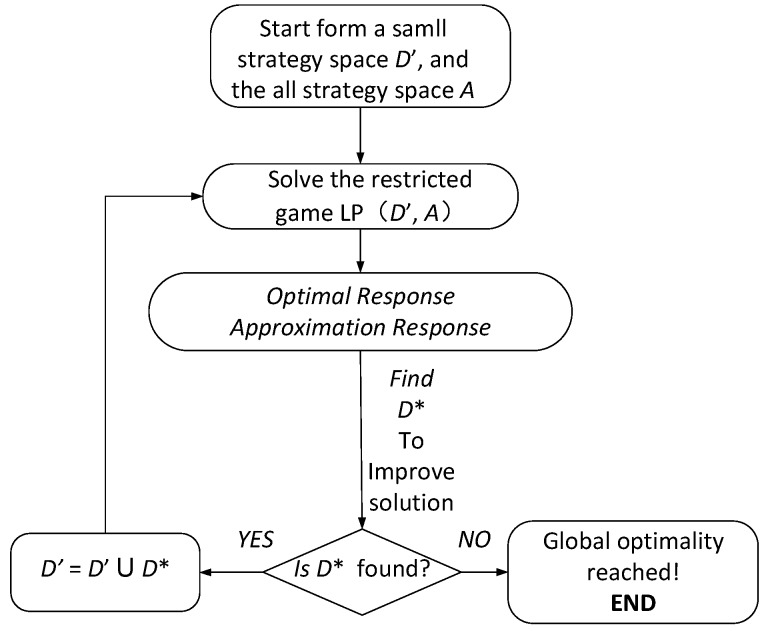
CG-AOD Framework.

An overall flow chart of the column generation algorithm is given in Figure 3. First, after giving the initial strategy, LP is utilized to solve the small-scale game equilibrium, and then loop iteratively to call the *Approximation Response* and the *Optimal Response*. It should be noted that the *Approximation Response* must be called first. If the *Approximation Response* cannot be found, the *Optimal Response* is called. When a better strategy for the defender is not found, the algorithm terminates. The final result converges to the global optimal solution.

The algorithm is described in detail using pseudo code as shown in Algorithm 1. CG-AOD is sketched in Algorithm 1. The first line initializes the strategies of the participating parties. The attacker’s initial strategy is to choose a random node as the source of infection, while defender randomly select *k* nodes as its initial strategy, i.e., $D^{\prime }=\{v|v\in V\}$. Equations (12)–(16) are used in line 5 to solve the equilibrium of both sides. A heuristic algorithm is utilized by *Approximation Response* to update strategies in line 6. *Approximation Response* utilizes Mixed Integer Linear Programming (MILP) to obtain the optimal strategy of the defender in line 8. The algorithm terminates when the optimal strategy cannot be found.
**Algorithm 1 Overview of CG-AOD****1:**Input: V,A,B;**2:**Output: the mixed strategies of both players (x,y);**3:**$Initialize\;D^{\prime },A;$**4: *repeat***;**5:**  $\left(x,y\right)\leftarrow \mathrm{CoreLP}\left(D^{\prime },A\right)$;**6:**   $D^{*}\leftarrow \mathrm{DAR}-D\left(y\right)$;**7:  *if***$D^{*}=\emptyset \;\mathrm{then};$**8:**    $D^{*}\leftarrow \mathrm{DOR}-D\left(y\right)$;**9:**    $D^{*}\leftarrow D^{\prime }\cup D^{*};$**10:**  $until\;D^{*}=\emptyset \;\mathrm{and}\;\;A^{*}=\emptyset$;11: return(x,y);

### 4.2. Optimal Response

The defender module consists of two parts: *Optimal Response* and *Approximation Response*. *Optimal Response*, which is responsible for obtaining the optimal response (i.e., optimal strategy) of the defender to the attacker’s mixed strategy, introduced in this section.

Given the attacker’s mixed strategy, the defender needs to find a pure strategy as its optimal response. When the defender’s strategy D satisfying $U_{d}\left(D,y\right)>U_{d}\left(x,y\right)$, the strategy is valid and we add it to the current strategy space $D^{\prime }$. The *Optimal Response* thus finds an improving strategy by minimizing $U_{d}\left(D,y\right)$ over the entire pure strategy space, and is formulated as the following MILP:

(17) $$max-\sum_{A\in A}y_{A}\left\{\frac{1}{MC}\sum_{i=0}^{MC-1}\sum_{j=0}^{N-1}\gamma_{j}^{A}\left(i\right)t_{j}^{A}\left(i\right)\right\}$$

(18) $$s.t.\;\;\sum_{v\in V}D_{v}=k$$

(19) $$\gamma_{j}^{A}\left(i\right)\leq D_{j}$$

(20) $$\beta_{j}^{A}\left(i\right)\leq \gamma_{j}^{A}\left(i\right)$$

(21) $$D_{j}t_{j}^{A}\left(i\right)-\left(1-\gamma_{j}^{A}\left(i\right)\right)M_{1}\leq \left(1-\beta_{j}^{A}\left(i\right)\right)M_{2}+D_{l}t_{l}^{A}\left(i\right)\;\forall j,l\in V$$

(22) $$D_{v}\in \left\{0,1\right\}$$

(23) $$\gamma_{j}^{A}\left(i\right)\in \left\{0,1\right\}$$

(24) $$\beta_{j}^{A}\left(i\right)\in \left\{0,1\right\}$$

(25) $$\sum_{i=0}^{MC-1}\gamma_{j}^{A}\left(i\right)=1\;for\;all\;A\;and\;i$$

(26) $$M_{1},\;M_{2}\;are\;big\;constants$$

Equation (17) is the optimization goal, where $t_{j}\left(i\right)$ is taken from the “Propagation Time Initialization Table”. Equation (18) enforces that the defender covers at most k nodes. Equations (19)–(25) ensure that the detection time of the outbreak propagation is minimal. Unfortunately, *Optimal Response* turns out to be NP-hard [44]. Therefore, to speed up the computational process, *Approximation Response* is required to find one pure strategy that can increase the payoff of defenders. It should be noted that it is not necessary to find the optimal solution in each iteration step, so the *Optimal Response* is called only when the *Approximation Response* cannot find such a strategy.

**Theorem** **1.** *The Optimal Response is NP-hard.*

**Proof** **1.** Proof of the *Optimal Response* can achieve by simplifying a Set-Cover Problem that is also NP-hard. The Set-Cover problem is defined as follows: Given a set of *n* elements U, and $Q\subseteq 2^{U}$ is the subset of U, the goal of the set cover problem is to find *k* subsets of Q which can cover all the elements of U. We turn an arbitrary set-cover problem into a *Optimal Response* problem as follows.

First, we build all the sensors as a network G=〈V,E〉. For each element i∈U, a node *v* labeled {i} is added to the network. For each non-single subset Q⊆Q, we add a node *v* to the network labeled as Q. The set described above is named as the label set denoted as l(v). For each i∈U, Q⊆Q, we add an edge between the node labeled as {i} and the node labeled as Q. Then, we construct the support set of the attacker’s mixed strategy, i.e., the set where pure strategy is non-zero. For each element i∈U, we construct a corresponding attacker’s pure strategy denoted $A_{i}=\left\{U_{i\in Q,Q\in Ql^{-1}\left(Q\right)}\right\}$, where $l^{-1}\left(Q\right)$ is the node labeled as Q. Then, this strategy is added to the support set. Therefore, the attacker’s support set contains |U| pure strategies.

**Figure 4:**
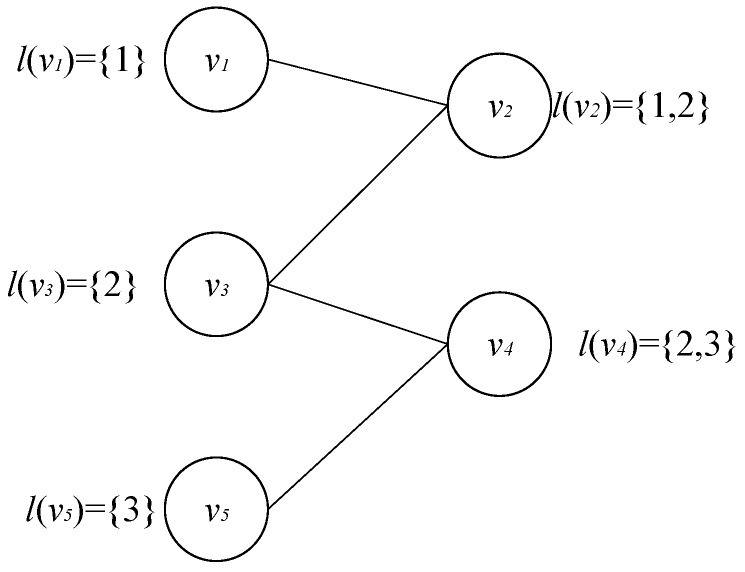
Constructed Graph.

For example, we assume that U = {1,2,3} and Q = {{1},{2},{3},{1,2},{2,3}}, constructed graph is shown as Figure 4.

The support set of attacker’s mixed strategy contains 3 pure strategies as shown in Table 2.

Then, we prove in the corresponding *Optimal Response* that the set U can be covered by *k* subsets in Q if and only if the defender can select all the attacker’s strategies. □

*Proof of sufficiency*: We assume that the defender can assign *k* resources to *k* nodes in the graph to monitor all the attacker’s strategies. *B* denotes the set of *k* nodes. It is proved that for ∀v∈B, the set U can be covered by *k* sets l(v).

We assume that the set U cannot be covered by kl(v) for arbitrary v∈B, which shows that there is at least one element i∈U to make i∉l(v). Note that the attacker’s strategy $A_{i}$ can only be the strategy with a non-zero probability. $A_{i}$ is defined as $\forall v\in A_{i}$, ∀i∈l(v). Thus nodes in $A_{i}$ are not contained in *B*, and $A_{i}$ is not monitored by *k* resources, which is contradict with the assumptions.

*Proof of necessity*: If the set U can be covered by *k* subsets Q⊆Q and defender can monitor all the attacker’s strategies by assigning *k* resources to nodes, then these nodes’ label sets are the *k* subsets of Q. The reason for that can be described as follows. For each {i}∈U, if there is a node *v* with i∈l(v) which is covered, then $A_{i}$ will be monitored. If U can be covered by *k* subsets Q, for each i∈U, it is at least one of the *k* subsets. Thus, all attacker’s strategies can be monitored by *k* resources.

### 4.3. Approximation Response

The previous section mainly introduces the *Optimal Response*, while this section focuses on another part called the *Approximation Response*. The *Approximation Response* is mainly responsible for finding strategies to improve the payoff of defenders. In this paper, the greedy algorithm is used to calculate the pure strategy for improving the payoff of defenders [49], i.e., $U_{d}\left(D,y\right)>U_{d}\left(x,y\right)$. First, we prove the payoff function is a sub-modularity function, which can get a good solution utilizing the greedy algorithm. A basic conclusion is drawn in literature [50]: for a submodular function, the greedy algorithm implements a constant factor approximation. At least one constant actor (1 − 1/*e*) can be obtained through the set of greedy algorithm D, which is based on the observation point value obtained by the optimal solution.

**Theorem** **2.** *The greedy algorithm ensures that the accuracy of Approximation Response is 1 − 1/e.*

**Proof** **2.** First, we define the normalized utility function as follows:

(27) $$ℱ\left(D\right)=U_{d}\left(D,y\right)-U_{d}\left(\emptyset ,y\right)=\underset{A\in y|A\cap D\neq \emptyset }{\sum }y_{a}P\left(A\right)$$

Next, we prove the function ℱ(D) is a submodule. Let $v_{1}$ and $v_{2}$ be two node sets chosen by the defender, and $v_{1}\subseteq v_{2}$. Let ${\bar{A}}_{1}$ and ${\bar{A}}_{2}$ be the set of attacker strategies that cannot be monitored by $v_{1}$ and $v_{2}$, i.e., ${\bar{A}}_{1}\in y|{\bar{A}}_{1}\cap v_{1}=\emptyset$ and ${\bar{A}}_{2}\in y|{\bar{A}}_{2}\cap v_{2}=\emptyset$. We have ${\bar{A}}_{2}\subseteq {\bar{A}}_{1}$ because any strategy monitored by $v_{1}$ can be monitored by $v_{2}$, thus ${\bar{A}}_{1}\subseteq {\bar{A}}_{2}$. As long as the function ℱ(D) is submodular, it must satisfy the equation as follows:

(28) $$ℱ\left(V_{1}\cup v\right)-ℱ\left(V_{1}\right)\geq ℱ\left(V_{2}\cup v\right)-ℱ\left(V_{2}\right)$$

Then we have:

(29) $$ℱ\left(V_{1}\cup v\right)-ℱ\left(V_{1}\right)=\sum_{A\in y|A\cap (V_{1}\cup v)\neq \emptyset }y_{a}P\left(A\right)-\sum_{A\in y|A\cap D\neq \emptyset }y_{a}P\left(A\right)=\sum_{A\in {\bar{A}}_{2}|A\cap v\neq \emptyset }y_{a}P\left(A\right)$$

For the right-hand side of Equation (28), we can also get:

(30) $$ℱ\left(V_{2}\cup v\right)-ℱ\left(V_{2}\right)=\underset{A\in {\bar{A}}_{2}|A\cap v\neq \emptyset }{\sum }y_{a}P\left(A\right)$$

Because ${\bar{A}}_{1}\subseteq {\bar{A}}_{2}$, $y_{a}P\left(A\right)>0$, Equation (28) is proved. Because ℱ(D) is submodular, the algorithm ensures the (1 − 1/e) accuracy. Pseudo-code is used to describe the greedy algorithm of the *Approximation Response* in detail, as shown in Algorithm 2.

As presented in Algorithm 2, the greedy algorithm tries to select a valid defender strategy iteratively. Input *y* is the attacker’s strategy space, but only the strategies with non-zero probabilities are considered. Defender’s strategy set $D_{appro}$ in line 3 is empty at the beginning, and activated sensors are added in $D_{appro}$ for every loop. Lines 4-8 are used for the update. Line 7 iteratively chooses the nodes that bring the largest marginal utility greedily. If no node satisfies the condition, nodes are randomly selected and then a measurement is generated in lines 9-10. Finally, it is required to judge whether the found strategy is valid. If it is valid, it will return, otherwise, it will return null.
**Algorithm 2 Approximation Response****1:**input:y ;**2:**output: D;**3:**$D_{appro}=\emptyset ;$**4:**for v∈V do;**5:**  D←{v};**6:**  while |D|<k do;**7:**    $v^{*}\leftarrow arg\;max_{v}U_{d}\left(D\cup \left\{v\right\},y\right)$;**8:**    $D\leftarrow D\cup \left\{v^{*}\right\};$**9:**while |D|<B do;**10:**      D←D∪{v} choose v from V randomly;**11:**   ***if***$\;U_{d}\left(D,y\right)>U_{d}\left(x,y\right)\;then;$**12:**     $D_{appro}=D_{appro}\cup \left\{D\right\}$;**13:**    $return\;D_{appro};$**14:**   ***else***;**15:**   ***return null***. □

## 5. Experimental Evaluation

In this section, we first evaluate the performance of our approach through a numerical example to demonstrate the validity of our approach in Section 4. Then, we test our algorithms on synthetic networks, and analyze the algorithms from the aspects of solution quality, scalability and robustness. All algorithms were run on a 3.20 GHz CPU loads with quad-cores and 16.00 GB memory using Windows 10. All linear programming involved is calculated using the solution software CPLEX (version 12.6). Since uncertainty is involved in the model, we run the simulation 100 times for obtaining each average result (the number 100 comes from the discussion “how many simulation runs are needed before obtaining an average result?” in [51]).

### 5.1. Numerical Example 

**Figure 5:**
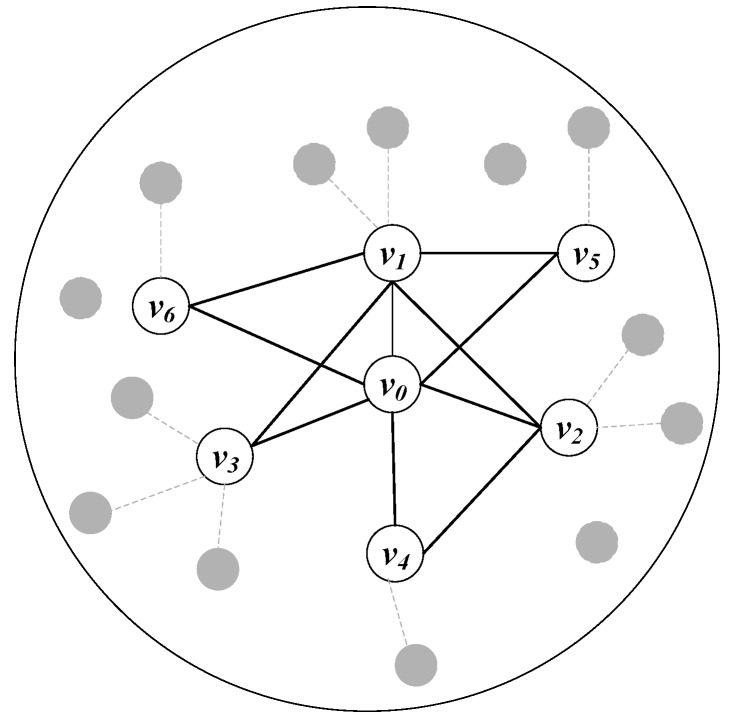
Small Network Graph.

In this part, we use a numerical example to verify the validity of the CG-AOD algorithm which included *Optimal Response* and *Approximation Response*. Given an undirected network G, as shown in Figure 5, the undirected graph has seven devices which are embedded with specific IDS. The solid line in the Figure 5 represents the link state of two devices, while the dotted line represents the unstable link relationship between devices. For simplicity, only seven nodes $V=\left\{v_{0},v_{1},\ldots ,v_{6}\right\}$ with stable links are selected for calculation. The label corresponding to each edge is represented as p=0.1. Among them, the maximum monitoring time is $T_{max}=10$, the budget of each defender strategy is k=3.

We calculate this numerical example by exploiting the mathematical programming in Equations (12)–(16), and the final exact solution is 2.78624. In the other words, the best time result of the detection by the defender for the outbreak propagation is 2.78624 seconds. We use the CG-AOD algorithm to solve the example given in Figure 5, and show the execution results of the algorithm and the process of CG-AOD in Table 3, which presents the nodes included in the two players’ strategy sets and the utility of the attackers in each time step.

When the time step is 1, it is the initialization strategy. Each time step loop calls the *Approximation Response* and *Optimal Response* to select defender strategy and calculates the Nash equilibrium of the two players under the current strategy.

### 5.2. Solution Quality Analysis 

In this section, we conduct experiments on synthetic graph and generate synthetic graphs from graph models: the Barabási-Albert, which is scale-free networks (BA(*r*)) and where each vertex is connected to *m* incumbents using a preferential attachment mechanism [52]. The distribution in the scale-free network satisfies the power law, which includes a large number of nodes with small degrees. Besides, the networks of different sizes can be generated by modifying parameters to evaluate the performance of the algorithm at different averaging. To establish a scale-free network, it is needed to be aware that when the new node *v* and the side of *v* exist are directed. We use BA scale-free network with parameters *r* = 2,4, labeled BA(2), and BA(4), respectively. For example, BA(2) indicates that there are two initial nodes, and each new node is connected with two existing nodes, thus forming a scale-free network. 

#### 5.2.1. Comparison of the Approximation Solution with the Optimal Solution

**Figure 6:**
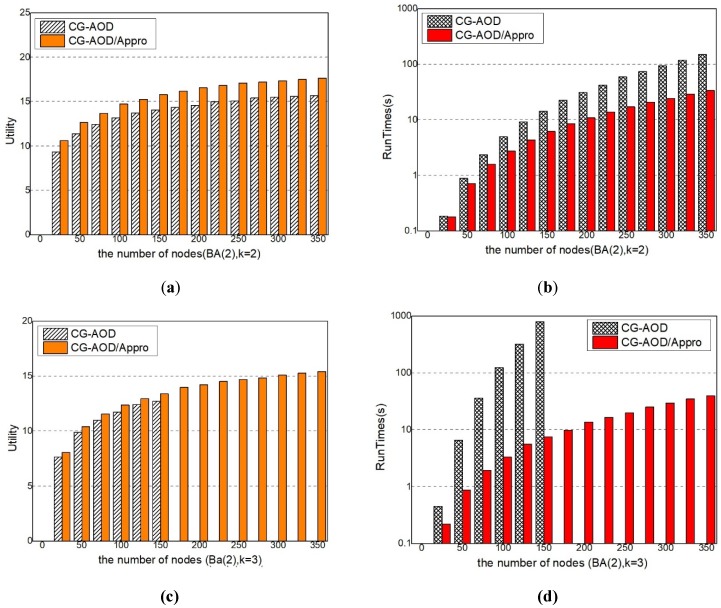

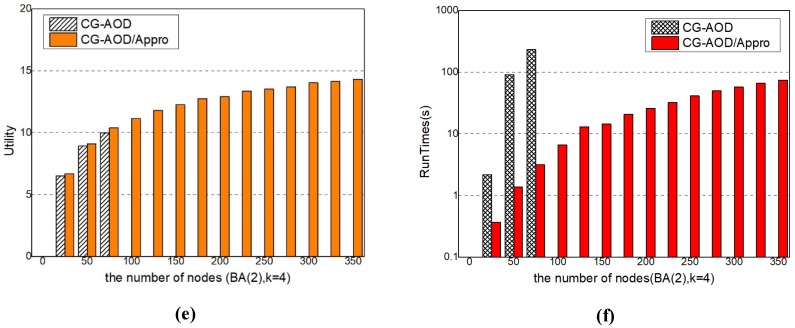
The Utility (**a**) and Runtime (**b**–**d**) of CG-AOD and CG-AOD/Appro (**e**,**f**).

*Approximation*. With the increase of network scale, the computational complexity increases, so that the results of CG-AOD algorithm cannot be obtained within a limited time. And thus we only run CG-AOD/Appro algorithm, which is one part of CG-AOD algorithm and without *Optimal Response*. In the BA(2) network, *k* = 2, we give the process of solving the global optimal solution directly using the mathematical programming in Equations (17)–(26) and compare CG-AOD/Appro algorithm with the CG-AOD algorithm. The experiment result shows that the approximate value of the solution quality of the CG-AOD/Appro algorithm and the CG-AOD algorithm is about 90%, as shown in Figure 6a. In the BA(2) network, *k* = 3, only a part of optimal solutions are obtained. As the number of sensors increases, the *Optimal Response* part will run out of resources in the calculation, so it cannot give all the optimal solutions. Nevertheless, it is not difficult to see that the approximate solution and the optimal solution given directly follow an approximate degree of more than 90%, which is higher than that at *k* = 2, as shown in Figure 6c. For BA(4), *k* = 4 has a similar result, as shown in Figure 6e. 

*Scalability Analysis.* We compare the scalability of the two methods under different resource numbers on BA(2) networks, as shown in Figure 6b–f. When the network and budget are relatively small, the difference of run time of CG-AOD and CG-AOD/Appro algorithm is little, but the CG-AOD/Appro algorithm performs better on large-scale networks, and can give the calculation results in a relatively short time. In Figure 6d,f, the complete solution of CG-AOD is not given, because the runtime of CG-AOD exponentially increases with the size. It is difficult to get results in a short time when the network size and budget are large. Overall, CG-AOD/Appro performs better than CG-AOD and can be scaled up to realistic-sized problems.

#### 5.2.2. Comparison of the CG-AOD/Appro Algorithm with Other Benchmark Methods

In this section, we choose six benchmark methods as a comparison to evaluate the solution quality of the CG-AOD/Appro algorithm in a complex network. 

*Benchmark methods.* We introduce three pure measure baselines of heuristic algorithms and three mixed measure baselines of heuristic algorithms. Different strategies of defender choosing in the benchmark are shown as follows:

- *RP*: the defender randomly opens *k* nodes for monitoring, and the attacker randomly selects a node as the source node of the infection.
- *DCP* [53]: the defender, according to the node degree value, selects the nodes to monitor, and then the attacker makes the *Optimal Response* according to the defender’s pure strategy.
- *CELF* [10]: the defender uses the CELF algorithm that is the classical greedy algorithm to choose *k* nodes for the sensor.
- *RM*: a defender mixed strategy where the marginal coverage probability of each vertex is a random value.
- *DCM*: a defender mixed strategy where the marginal coverage probability of each vertex is normalized degree centrality.

**Figure 7:**
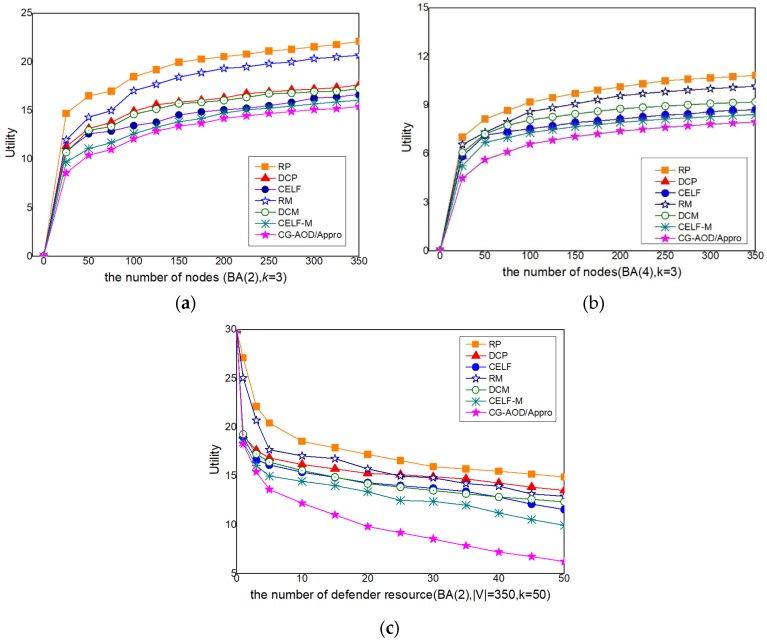
The performance of CG-AOD/Appro on synthetic networks against attackers (**a**–**c**).

*CELF-M*: a defender mixed strategy where the marginal coverage probability of each vertex is normalized CELF value-based centrality. *Solution Quality Analysis*. In Figure 7a–b, we compare the solution quality obtained from our approaches with those obtained from 6 heuristic baselines. Results show that the solutions of all the heuristic baselines are not ideal in general and become worse with the increasing network size, while CG-AOD/Appro can always achieve an almost optimal solution, which is the closest to the solution given by CG-AOD. The performance of RP is dramatically worse in all cases because it did not consider the network structure. The performance of all mixed strategy heuristics (i.e., RM, DCM, and CELF-M) is much better than the corresponding pure strategy-based heuristics (i.e., RP, DCP, and CELF), confirming the necessity of using randomized dynamic monitoring schedules. The good performance of CG-AOD/Appro indicates that our scheme has a better effective response.

*Robustness*. We compare the utility of CG-AOD/Appro under different the number of defender resources. Figure 7c shows a decreasing efficiency of CG-AOD/Appro which is faster than the other 6 heuristic baselines. The solutions are still near-optimal and outperform other heuristic algorithms significantly.

The above experiments show that the solutions of all the baselines are not ideal in general and become worse with the increasing network size when the defender strategy obtained from the six baselines, while CG-AOD/Appro can always obtain almost the optimal solution. CG-AOD/Appro offers significant performance and robustness.

## 6. Conclusions

In this paper, we investigate the problem of adversarial outbreak detection (AOD) in the IoT, in which a network defender aims to detect the attacker as soon as possible by generating a dynamic scheduling strategy in the network. There are two key issues to be considered: (1) IoT devices usually have limited resources and the sensors cannot detect the outbreak propagation continuously; (2) the IoT devices are usually expose to the public situation, and the attacker can strategically employ analytical tools to maximize the influence of worms’ propagation. We present the work through applying Stackelberg security games for outbreak detection in the adversarial environment and study the strategies and utility of both players. Firstly, we formulate the interaction between the defender and the attacker as a Stackelberg game. Secondly, we build a modified Column Generation framework for computing the optimal strategy effectively and construct the optimal response of defenders as a mixed-integer linear programming (MILPs), and prove that solving the optimal response of defenders is an NP-hard problem. Then, the optimal response of defenders are improved by using an approximate algorithm-greedy algorithm. Finally, we demonstrate the effectiveness, scalability and robustness of our scheme through experiments.

## Figures and Tables

**Table 1:** Propagation Time Initialization Table.

Node	$v_{0}$	$v_{1}$	…	$v_{j}$	…	$v_{n}$
$u_{0}$	0	α(0,1)	…	α(0,j)	…	α(0,n)
$u_{1}$	α(1,0)	0	…	α(1,j)	…	α(1,n)
…	…	…	…	…	…	…
$u_{i}$	α(i,0)	α(i,1)	…	α(i,j)	…	α(i,n)
…	…	…	…	…	…	…
$u_{n}$	α(n,0)	α(n,1)	…	α(n,j)	…	0

**Table 2:** Mixed Strategy.

Pure Strategy	Corresponding Element in U	Vertices
$A_{1}$	1	$\left\{v_{1},v_{2}\right\}$
$A_{2}$	2	$\left\{v_{2},v_{3},v_{4}\right\}$
$A_{3}$	3	$\left\{v_{4},v_{5}\right\}$

**Table 3:** The Process of CG-AOD.

STEP	Attacker Strategy	Defender Strategy	Payoff
1	$\left\{v_{0}\right\},\left\{v_{1}\right\},\left\{v_{2}\right\},\left\{v_{3}\right\},\left\{v_{4}\right\},\left\{v_{5}\right\},\left\{v_{6}\right\}$	{$v_{4}$,$v_{5}$,$v_{3}$}	
Equilibrium	$\left\{v_{6}\right\}\left(1\right)$	{$v_{4}$,$v_{5}$,$v_{3}$}(1)	9
2	$\left\{v_{0}\right\},\left\{v_{1}\right\},\left\{v_{2}\right\},\left\{v_{3}\right\},\left\{v_{4}\right\},\left\{v_{5}\right\},\left\{v_{6}\right\}$	{$v_{4}$,$v_{5}$,$v_{3}$},{$v_{4}$,$v_{5}$,$v_{6}$}	
Equilibrium	$\left\{v_{2}\right\}$(0.891089) $\left\{v_{6}\right\}$(0.108911)	{$v_{4}$,$v_{5}$,$v_{3}$}(0.613861) {$v_{4}$,$v_{5}$,$v_{6}$}(0.386139)	5.52475
3	$\left\{v_{0}\right\},\left\{v_{1}\right\},\left\{v_{2}\right\},\left\{v_{3}\right\},\left\{v_{4}\right\},\left\{v_{5}\right\},\left\{v_{6}\right\}$	{$v_{4}$,$v_{5}$,$v_{3}$},{$v_{4}$,$v_{5}$,$v_{6}$}, {$v_{2}$,$v_{5}$,$v_{6}$}	
Equilibrium	$\left\{v_{3}\right\}$(0.473684) $\left\{v_{6}\right\}$(0.526316)	{$v_{4}$,$v_{5}$,$v_{6}$} (0.526316) {$v_{2}$,$v_{5}$,$v_{6}$}(0.473684)	4.73684
4	$\left\{v_{0}\right\},\left\{v_{1}\right\},\left\{v_{2}\right\},\left\{v_{3}\right\},\left\{v_{4}\right\},\left\{v_{5}\right\},\left\{v_{6}\right\}$	{$v_{4}$,$v_{5}$,$v_{3}$},{$v_{4}$,$v_{5}$,$v_{6}$}, {$v_{2}$,$v_{5}$,$v_{6}$},{$v_{3}$,$v_{5},v_{6}$}	
Equilibrium	$\left\{v_{0}\right\}$(0.99099) $\left\{v_{3}\right\}$(0.00990099)	{$v_{4}$,$v_{5}$,$v_{3}$}(0.418042) {$v_{2}$,$v_{5}$,$v_{6}$}(0.376238) {$v_{3}$,$v_{5},v_{6}$}(0.205721)	3.76238
5	$\left\{v_{0}\right\},\left\{v_{1}\right\},\left\{v_{2}\right\},\left\{v_{3}\right\},\left\{v_{4}\right\},\left\{v_{5}\right\},\left\{v_{6}\right\}$	{$v_{4}$,$v_{5}$,$v_{3}$},{$v_{4}$,$v_{5}$,$v_{6}$}, {$v_{2}$,$v_{5}$,$v_{6}$},{$v_{3}$,$v_{5},v_{6}$}, $\{v_{0}$,$v_{3}$,$v_{6}$}	
Equilibrium	$\left\{v_{1}\right\}$(0.898204) $\left\{v_{3}\right\}$(0.0718563) $\left\{v_{6}\right\}$(0.0299401)	{$v_{4}$,$v_{5}$,$v_{3}$}(0.409182) {$v_{2}$,$v_{5}$,$v_{6}$}(0.368263) {$v_{0}$,$v_{3}$,$v_{6}$}(0.222555)	3.68263
6	$\left\{v_{0}\right\},\left\{v_{1}\right\},\left\{v_{2}\right\},\left\{v_{3}\right\},\left\{v_{4}\right\},\left\{v_{5}\right\},\left\{v_{6}\right\}$	{$v_{4}$,$v_{5}$,$v_{3}$},{$v_{4}$,$v_{5}$,$v_{6}$}, {$v_{2}$,$v_{5}$,$v_{6}$},{$v_{3}$,$v_{5},v_{6}$}, {$v_{0}$,$v_{3}$,$v_{6}$},{$v_{1}$,$v_{3}$,$v_{6}$}	
Equilibrium	$\left\{v_{1}\right\}$(0.0539423) $\left\{v_{2}\right\}$(0.552908) $\left\{v_{3}\right\}$(0.33606) $\left\{v_{6}\right\}$(0.0570889)	{$v_{4}$,$v_{5}$,$v_{3}$}(0.393179) {$v_{2}$,$v_{5}$,$v_{6}$}(0.353861) {$v_{3}$,$v_{5}$,$v_{6}$}(0.213851) {$v_{1}$,$v_{3}$,$v_{6}$}(0.0391082)	3.53861
7	$\left\{v_{0}\right\},\left\{v_{1}\right\},\left\{v_{2}\right\},\left\{v_{3}\right\},\left\{v_{4}\right\},\left\{v_{5}\right\},\left\{v_{6}\right\}$	{$v_{4}$,$v_{5}$,$v_{3}$},{$v_{4}$,$v_{5}$,$v_{6}$}, {$v_{2}$,$v_{5}$,$v_{6}$},{$v_{3}$,$v_{5}$,$v_{6}$}, {$v_{0}$,$v_{3}$,$v_{6}$},{$v_{1}$,$v_{3}$,$v_{6}$}, $\{v_{2}$,$v_{3}$,$v_{6}$}	
Equilibrium	$\left\{v_{0}\right\}$(0.239721) $\left\{v_{1}\right\}\left(0.421699\right)$ $\left\{v_{2}\right\}$(0.129297) $\left\{v_{3}\right\}$(0.0127931) $\left\{v_{4}\right\}$(0.196489)	{$v_{4}$,$v_{5}$,$v_{6}$}(0.275675) {$v_{2}$,$v_{5}$,$v_{6}$}(0.317281) $\{v_{0}$,$v_{3}$,$v_{6}$}(0.150301) {$v_{1}$,$v_{3}$,$v_{6}$}(0.135167) {$v_{2}$,$v_{3}$,$v_{6}$}(0.121576)	3.17281
8	$\left\{v_{0}\right\},\left\{v_{1}\right\},\left\{v_{2}\right\},\left\{v_{3}\right\},\left\{v_{4}\right\},\left\{v_{5}\right\},\left\{v_{6}\right\}$	{$v_{4}$,$v_{5}$,$v_{3}$},{$v_{4}$,$v_{5}$,$v_{6}$}, {$v_{2}$,$v_{5}$,$v_{6}$},{$v_{3}$,$v_{5},v_{6}$}, $\{v_{0}$,$v_{3}$,$v_{6}$},{$v_{1}$,$v_{3}$,$v_{6}$}, {$v_{2}$,$v_{3}$,$v_{6}$},{$v_{0}$,$v_{1}$,$v_{4}$}	
Equilibrium	$\left\{v_{0}\right\}$(0.175687) $\left\{v_{1}\right\}$(0.183212) $\left\{v_{2}\right\}$(0.208893) $\left\{v_{3}\right\}$(0.17239) $\left\{v_{5}\right\}$(0.198778) $\left\{v_{6}\right\}$(0.061041)	{$v_{4}$,$v_{5}$,$v_{3}$}(0.268945) {$v_{2}$,$v_{5}$,$v_{6}$}(0.239261) $\{v_{0}$,$v_{3}$,$v_{6}$}(0.106774) {$v_{1}$,$v_{3}$,$v_{6}$}(0.100118) {$v_{2}$,$v_{3}$,$v_{6}$}(0.191897) {$v_{0}$,$v_{1}$,$v_{4}$}(0.0930045)	2.97854
9	$\left\{v_{0}\right\},\left\{v_{1}\right\},\left\{v_{2}\right\},\left\{v_{3}\right\},\left\{v_{4}\right\},\left\{v_{5}\right\},\left\{v_{6}\right\}$	{$v_{4}$,$v_{5}$,$v_{3}$},{$v_{4}$,$v_{5}$,$v_{6}$}, {$v_{2}$,$v_{5}$,$v_{6}$},{$v_{3}$,$v_{5},v_{6}$}, $\{v_{0}$,$v_{3}$,$v_{6}$},{$v_{1}$,$v_{3}$,$v_{6}$}, {$v_{2}$,$v_{3}$,$v_{6}$},{$v_{0}$,$v_{1}$,$v_{4}$}, $\{v_{2}$,$v_{3}$,$v_{5}$}	
Equilibrium	$\left\{v_{0}\right\}$(0.01404) $\left\{v_{1}\right\}$(0.211501) $\left\{v_{2}\right\}$(0.0971293) $\left\{v_{3}\right\}$(0.140188) $\left\{v_{4}\right\}$(0.198705) $\left\{v_{5}\right\}$(0.163512) $\left\{v_{6}\right\}$(0.174924)	{$v_{4}$,$v_{5}$,$v_{6}$} (0.22779) {$v_{2}$,$v_{5}$,$v_{6}$}(0.236859) {$v_{0}$,$v_{3}$,$v_{6}$}(0.121694) {$v_{1}$,$v_{3}$,$v_{6}$} (0.113244) $\{v_{2}$,$v_{3}$,$v_{6}$}(0.171224) {$v_{0}$,$v_{1}$,$v_{4}$} (0.088595) {$v_{2}$,$v_{3}$,$v_{5}$}(0.0405945)	2.92673
10	$\left\{v_{0}\right\},\left\{v_{1}\right\},\left\{v_{2}\right\},\left\{v_{3}\right\},\left\{v_{4}\right\},\left\{v_{5}\right\},\left\{v_{6}\right\}$	{$v_{4}$,$v_{5}$,$v_{3}$},{$v_{4}$,$v_{5}$,$v_{6}$}, {$v_{2}$,$v_{5}$,$v_{6}$},{$v_{3}$,$v_{5},v_{6}$}, {$v_{0}$,$v_{3}$,$v_{6}$},{$v_{1}$,$v_{3}$,$v_{6}$}, {$v_{2}$,$v_{3}$,$v_{6}$},{$v_{0}$,$v_{1}$,$v_{4}$}, $\{v_{2}$,$v_{3}$,$v_{5}$},{$v_{1}$,$v_{3}$,$v_{4}$}	
Equilibrium	$\left\{v_{0}\right\}$(0.00326235) $\left\{v_{1}\right\}$(0.0742531) $\left\{v_{2}\right\}$(0.122845) $\left\{v_{3}\right\}$(0.169188) $\left\{v_{4}\right\}$(0.215608) $\left\{v_{4}\right\}$(0.2072) $\left\{v_{6}\right\}$(0.174924)	{$v_{4}$,$v_{5}$,$v_{3}$} (0.0268422) {$v_{2}$,$v_{5}$,$v_{6}$} (0.278986) $\{v_{0}$,$v_{3}$,$v_{6}$}(0.0251817) $\{v_{2}$,$v_{3}$,$v_{6}$}(0.056574) $\{v_{2}$,$v_{3}$,$v_{5}$}(0.134177) {$v_{1}$,$v_{3}$,$v_{4}$} (0.230782)	2.78986
11	$\left\{v_{0}\right\},\left\{v_{1}\right\},\left\{v_{2}\right\},\left\{v_{3}\right\},\left\{v_{4}\right\},\left\{v_{5}\right\},\left\{v_{6}\right\}$	{$v_{4}$,$v_{5}$,$v_{3}$},{$v_{4}$,$v_{5}$,$v_{6}$}, {$v_{2}$,$v_{5}$,$v_{6}$},{$v_{3}$,$v_{5},v_{6}$}, $\{v_{0}$,$v_{3}$,$v_{6}$},{$v_{1}$,$v_{3}$,$v_{6}$}, $\{v_{2}$,$v_{3}$,$v_{6}$},{$v_{0}$,$v_{1}$,$v_{4}$}, {$v_{2}$,$v_{3}$,$v_{5}$},{$v_{1}$,$v_{3}$,$v_{4}$} {$v_{3}$,$v_{4}$,$v_{6}$}	
Equilibrium	$\left\{v_{0}\right\}$(0.0176406) $\left\{v_{1}\right\}\left(0.103868\right)$ $\left\{v_{2}\right\}$(0.124691) $\left\{v_{3}\right\}$(0.165048) $\left\{v_{4}\right\}$(0.186597) $\left\{v_{5}\right\}$(0.199553) $\left\{v_{6}\right\}$(0.202604)	{$v_{2}$,$v_{5}$,$v_{6}$}(0.278624) {$v_{0}$,$v_{3}$,$v_{6}$} (0.0248471) {$v_{0}$,$v_{3}$,$v_{6}$}(0.246089) $\{v_{2}$,$v_{3}$,$v_{6}$}(0.029415) {$v_{2}$,$v_{3}$,$v_{5}$} (0.162656) {$v_{1}$,$v_{3}$,$v_{4}$} (0.230108) {$v_{3}$,$v_{4}$,$v_{6}$}(0.0282604)	2.78624

## References

[1] Li S., Xu L., Zhao S. (2015). The Internet of Things: A Survey.

[2] Alaba F.A., Othman M., Hashem I.A.T., Alotaibi F. (2017). Internet of things Security: A Survey. J. Netw. Comput. Appl..

[3] Wang T., Wu Q., Wen S., Cai Y., Tian H., Chen Y., Wang B. (2017). Propagation Modeling and Defending of a Mobile Sensor Worm in Wireless Sensor and Actuator Networks. Sensors.

[4] Kolias C., Kambourakis G., Stavrou A., Jeffrey M.S. (2017). DDoS in the IoT. Mirai and Other Botnets. IEEE Comput..

[5] Kaspersky. https://www.kaspersky.com/blog/attack-on-dyn-explained/13325/.

[6] Csoonline. https://www.csoonline.com/article/3227906/what-is-wannacry-ransomware-how-does-it-infect-and-who-was-responsible.html.

[7] ZDNet. https://www.zdnet.com/article/iot-security-warning-cyber-attacks-on-medical-devices-could-put-patients-at-risk/.

[8] Zarpelao B.B., Miani R.S., Kawakani C.T., de Alvarenga S.C. (2017). A survey of intrusion detection in Internet of Things. J. Netw. Comput. Appl..

[9] Sharma S., Kaul A. (2018). A survey on Intrusion Detection Systems and Honeypot based proactive security mechanisms in VANETs and VANET Cloud. Veh. Commun..

[10] Leskovec J., Krause A., Guestrin C., Faloutsos C., Faloutsos C., VanBriesen J., Glance N. Cost-effective outbreak detection in networks. Proceedings of the 13th ACM SIGKDD International Conference on Knowledge Discovery and Data Mining.

[11] Krause A., Guestrin C. (2009). Optimizing Sensing: From Water to the Web. IEEE Comput..

[12] Krause A., Rajagopal R., Gupta A., Guestrin C. (2011). Simultaneous optimization of sensor placements and balanced schedules. IEEE Trans. Automat. Contr..

[13] Sela P.L., Abbas W., Koutsoukos X., Amin S. (2016). Sensor placement for fault location identification in water networks: A minimum test cover approach. Automatica.

[14] Jung D., Kim J.H. (2018). Using mechanical reliability in multiobjective optimal meter placement for pipe burst detection. J. Water Resour. Plan. Manag..

[15] Shakarian P., Lei H., Lindelauf R. Power grid defense against malicious cascading failure. Proceedings of the 2014 International Conference on Autonomous Agents and Multi-Agent Systems.

[16] Lin F.H., Zhou Y., An X., You I., Choo K.K.R. (2018). Fair Resource Allocation in an Intrusion-Detection System for Edge Computing: Ensuring the Security of Internet of Things Devices. IEEE Consum. Electron. Mag..

[17] Xu Q., Ren P., Song H., Du Q. (2016). Security enhancement for IoT communications exposed to eavesdroppers with uncertain locations. IEEE Access.

[18] Veerappan C.S., Keong P.L.K., Tang Z., Tan F. Taxonomy on malware evasion countermeasures techniques. Proceedings of the IEEE World Forum on Internet of Things.

[19] Hu H., Wang M., Ouyang M., Hu G. (2019). Toward Network Worm Victims Identification Based on Cascading Motif Discovery. Electronics.

[20] Bethencourt J., Franklin J., Vernon M.K. Mapping Internet Sensors with Probe Response Attacks. Proceedings of the USENIX Security Symposium.

[21] Hu F. (2016). Security and privacy in Internet of things (IoTs): Models, Algorithms, and Implementations.

[22] Zhou C., Lu W.X., Zhang J., Li L., Hu Y., Guo L. (2018). Early detection of dynamic harmful cascades in large-scale networks. J. Comput. Sci-Neth..

[23] Acarali D., Rajarajan M., Komninos N., Zarpelão B.B. (2019). Modelling the Spread of Botnet Malware in IoT-Based Wireless Sensor Networks. Secur. Commun. Netw..

[24] Sedjelmaci H., Senouci S.M., Al-Bahri M. A lightweight anomaly detection technique for low-resource IoT devices: A game-theoretic methodology. Proceedings of the IEEE International Conference on Communications.

[25] Liu B., Xu H., Zhou X. (2018). Stackelberg Dynamic Game-Based Resource Allocation in Threat Defense for Internet of Things. Sensors.

[26] Sohail M., Khan S., Ahmad R., Singh D., Lloret J. (2019). Game Theoretic Solution for Power Management in IoT-Based Wireless Sensor Networks. Sensors.

[27] Krause A., Guestrin C. (2011). Submodularity and its Applications in Optimized Information Gathering. ACM TIST.

[28] Huang C.T., Sakib M.N., Njilla L., Kamhoua C. A Game Theoretic Approach for Making IoT Device Connectivity Decisions During Malware Outbreak. Proceedings of the International Conference on Computing, Networking and Communications.

[29] Robert C., George C. (2013). Monte Carlo Statistical Methods. https://books.google.com.hk/books?hl=en&lr=&id=lrvfBwAAQBAJ&oi=fnd&pg=PR17&dq=Monte+Carlo+statistical+methods&ots=GNEp5duzJ5&sig=Oe3pCPujDaKSK25qQ4eoT_OrKDU&redir_esc=y&hl=zh-CN&sourceid=cndr#v=onepage&q=Monte%20Carlo%20statistical%20methods&f=false.

[30] Benkhelifa E., Welsh T., Hamouda W. (2018). A critical review of practices and challenges in intrusion detection systems for IoT: Toward universal and resilient systems. IEEE Commun. Suvr. Tut..

[31] Sforzin A., Mármol F.G., Conti M., Bohli J.M. RPiDS: Raspberry Pi IDS—A Fruitful Intrusion Detection System for IoT. Proceedings of the IEEE Conferences on Ubiquitous Intelligence & Computing, Advanced and Trusted Computing, Scalable Computing and Communications, Cloud and Big Data Computing, Internet of People, and Smart World Congress.

[32] Peng Y., Yang J., Wu C., Guo C., Hu C., Li Z. deTector: A Topology-aware Monitoring System for Data Center Networks. Proceedings of the USENIX Annual Technical Conference.

[33] Yu Y., Xiao G. (2014). On early detection of strong infections in complex networks. J. Phys. A-Math. Thero..

[34] Zhang H., Alim M.A., Thai M.T., Nguyen H.T. Monitor placement to timely detect misinformation in Online Social Networks. Proceedings of the IEEE International Conference on Communications.

[35] Krause A., McMahan B., Guestrin C., Gupta A. Selecting Observations against Adversarial Objectives. Proceedings of the Advances in Neural Information Processing Systems.

[36] Semasinghe P., Maghsudi S., Hossain E. (2017). Game theoretic mechanisms for resource management in massive wireless IoT systems. IEEE Commun. Mag..

[37] Rullo A., Midi D., Serra E., Bertino E. (2017). Pareto optimal security resource allocation for Internet of Things. TOPS.

[38] Altman E., Avrachenkov K., Garnaev A. Jamming in wireless networks: The case of several jammers. Proceedings of the 2009 International Conference on Game Theory for Networks.

[39] Namvar N., Saad W., Bahadori N., Kelley B. Jamming in the Internet of Things: A Game-Theoretic Perspective. Proceedings of the IEEE Global Communications Conference.

[40] Zhu Q., Bushnell L., Başar T. Game-theoretic analysis of node capture and cloning attack with multiple attackers in wireless sensor networks. Proceedings of the 51th IEEE Conference on Decision and Control.

[41] Cheng H.T., Zhuang W. (2009). Pareto optimal resource management for wireless mesh networks with QoS assurance: joint node clustering and subcarrier allocation. IEEE T. Wirel. Commun..

[42] Tsai J., Nguyen T.H., Tambe M. Security Games for Controlling Contagion. Proceedings of the 26th AAAI Conference on Artificial Intelligence.

[43] Yin Y., An B., Jain M. Game-theoretic resource allocation for protecting large public events. Proceedings of the 28th AAAI Conference on Artificial Intelligence.

[44] Wang Z., Yin Y., An B. Computing Optimal Monitoring Strategy for Detecting Terrorist Plots. Proceedings of the of the 30th AAAI Conference on Artificial Intelligence.

[45] Jain M., Kardes E., Kiekintveld C., Ordónez F., Tambe M. Security Games with Arbitrary Schedules: A Branch and Price Approach. Proceedings of the 24th AAAI Conference on Artificial Intelligence.

[46] Jain M., Kardes E., Kiekintveld C., Ordónez F., Tambe M. Security games with protection externalities. Proceedings of the 29th AAAI Conference on Artificial Intelligence.

[47] Hu X., Zhu W., An B., Jin P., Xia W. (2019). A branch and price algorithm for EOS constellation imaging and downloading integrated scheduling problem. Comput & OR.

[48] Rullo A., Serra E., Bertino E., Lobo J. Shortfall-based optimal placement of security resources for mobile IoT scenarios. Proceedings of the European Symposium on Research in Computer Security.

[49] Serra E., Jajodia S., Pugliese A., Rullo A., Subrahmanian V.S. (2015). Pareto-Optima Adversarial Defense of Enterprise Systems. ACM Tans. Inf. Syst. Secur..

[50] Nemhauser G.L., Wolsey L.A., Fisher M.L. (1978). An analysis of approximations for maximizing submodular set functions—I. Math. Program..

[51] Wen S., Zhou W., Zhang J., Xiang Y., Zhou W., Jia W., Zou C.C. (2014). Modeling and analysis on the propagation dynamics of modern email malware. IEEE Trans. Dependable. Secure. Comput..

[52] Barabási A.L., Albert R. (1999). Emergence of scaling in random networks. Science.

[53] Tsai J., Yin Z., Kwak J.Y., Kempe D., Kiehintveld C., Tambe M. (2010). Urban Security: Game-Theoretic Resource Allocation in Networked Domains. Proceedings of the 24th AAAI Conference on Artificial Intelligence.

